# Surface coating of orthopedic implant to enhance the osseointegration and reduction of bacterial colonization: a review

**DOI:** 10.1186/s40824-022-00269-3

**Published:** 2022-06-20

**Authors:** Smriti Bohara, Jackrit Suthakorn

**Affiliations:** Department of Biomedical Engineering, Center for Biomedical and Robotics Technology (BART LAB), Faculty of Engineering, Mahidol University, Salaya, Thailand

**Keywords:** Orthopedic implant, Infection, Coating, Osseointegration, Hydrogels, Antibiotics

## Abstract

The use of orthopedic implants in surgical technology has fostered restoration of physiological functions. Along with successful treatment, orthopedic implants suffer from various complications and fail to offer functions correspondent to native physiology. The major problems include aseptic and septic loosening due to bone nonunion and implant site infection due to bacterial colonization. Crucial advances in material selection in the design and development of coating matrixes an opportunity for the prevention of implant failure. However, many coating materials are limited in *in-vitro* testing and few of them thrive in clinical tests. The rate of implant failure has surged with the increasing rates of revision surgery creating physical and sensitive discomfort as well as economic burdens. To overcome critical pathogenic activities several systematic coating techniques have been developed offering excellent results that combat infection and enhance bone integration. This review article includes some more common implant coating matrixes with excellent in vitro and in vivo results focusing on infection rates, causes, complications, coating materials, host immune responses and significant research gaps. This study provides a comprehensive overview of potential coating technology, with functional combination coatings which are focused on ultimate clinical practice with substantial improvement on in-vivo tests. This includes the development of rapidly growing hydrogel coating techniques with the potential to generate several accurate and precise coating procedures.

## Introduction

Orthopedic implants are an indispensable part of medical treatment, and are surgically implanted in the human body to restore physiological functions. Implants replace and support fractured bone, bone unions, regeneration and also enhance mechanical stabilization [1]. In addition, implants are widely used in the treatment of fracture fixation, osteoarthritis, spinal deformation, knee, total hip replacement and other orthopedic related fixations. There is a clear correlation between the ageing population and implant surgery [2]. Every year millions of people go through bone implants for total hip and knee replacement. These include procedures like open or closed fracture fixation. Other implants include for scoliosis, maxillofacial fixation, and traumatic conditions.

Despite biological and engineering design modifications, sterilized operating room environments and regular antimicrobial prophylaxis [2] multidrug-resistant pathogens are increasing [3]. According to the “Third American Joint Replacement Registry (AJRR) Annual Report on Hip and Knee “Arthroplasty Data 2016”, there is a 10.2% increase in surgical procedures compared to previous years [4]. Orthoperiodic implants, when implanted in the host, are highly susceptible to bacteria due to the host immune fade zone. It takes only a few hours for microbial adhesion and bacterial colonization on the implant surface [5]. Bacteria have diversified strategies to adhere both to natural and synthetic surfaces with higher survival rates [6, 7]. Microbial infection is relatively higher in open fracture fixation than closed fixation [8], with the risk rates varying between 13.6% and 8% [9] respectively. Implant failure due to bacterial adhesion to the solid surface of the implant is followed by the development of a medium called biofilm [10]. Biofilms on orthopedic prosthesis are mainly due to *Staphylococcus aureus 20-30%* and *coagulase-negative Staphylococci 20–40*% [7, 10, 11], resulting in infection and failure of tissue integration. Especially with arthroplasties, biofilm formation and periprosthetic infections range from 1–9% depending on the type of arthroplasties: about 1% in hip and shoulder prostheses, 2% in knee prosthesis and 9% in elbow prostheses [12]. Spinal infections range from 2-5% [13]. The implant device infection ratio extends from 5% with an infection rate in external fixation, up to 30% [11]. AJRR reports from 2012 to 2015, that there were 169,060 hip arthroplasty procedures in the United States of America, of which 17,180 had revision surgery and 258,121 went for knee arthroplasty, among which 22,403 had revision surgery [4]. Consequently, this increased the revision surgical burden in patients by 10.2% and 8.7% for hip and knee respectively [14, 15]. The economic burden for this revision surgery due to prosthetic joint infection is increasing every year. The predicted hospital costs in the U.S. alone are over $500 million, which is anticipated to increase to $1.62 billion by 2030 [16].

Regarding the control of the increasing issues related to orthopedic implant revision surgery leading to implant failure, numerous techniques have been developed including engineering modification of implants, selection of implant materials, oral intake of antibiotics, coating of the implant with natural or synthetic polymeric hydrogel matrix, antibiotic coating and many other traditional and novel procedures. This review article however, includes some of the general coating techniques, used clinically for the reduction of surgical site infection and enhancing osseointegration. The paper aims to outline coating techniques to enhance bone integration, like hydroxyapatite, extracellular matrix/collagen, and magnesium coatings. The paper also aims to describe techniques for reduction of infection, such as direct antimicrobial coating, drug-loaded hydrogel coating and advanced combinatorial drug coating on implants, along with the associated drawbacks of the coating systems and finally, concludes with a discussion of future directions.

### Associative orthopedic implant-related complication

Despite the numerous applications and advances in treatment, orthopedic implants still suffer from complications and fail to offer functions with respect to the native physiological structure [17]. Among these, “bone non-union” and infection are leading causes of revision surgery and implant failure. “Bone non-union” is acknowledged as septic and aseptic loosening in medical terms, where resistance at articulating surface or repetitive mechanical stress associated with locomotion occurs in cemented implants [18]. However, osseointegration is a key cause of failure of loosening in non-cemented implants [19]. Implant associated infection and loosening are responsible for 40-50% of total knee replacement revision surgeries every year [20, 21]. Total hip replacement implant loosening and infection, leading to device revision surgery is approximately 35% [20], which is comparatively lower than early revision surgery, which was 50% [22]. The second major complication with regards to bacterial inflammation: bacterial attachment and colonization on the orthopedic implant surface governing acute and chronic contagion of implant surrounding cells and tissues [23]. Infection caused by biofilm formation on the implant sites is a major problem related to implant failure, where post-operative infection in the implant site is significant and includes bone and joint degeneration [10, 17].

**Fig. 1:**
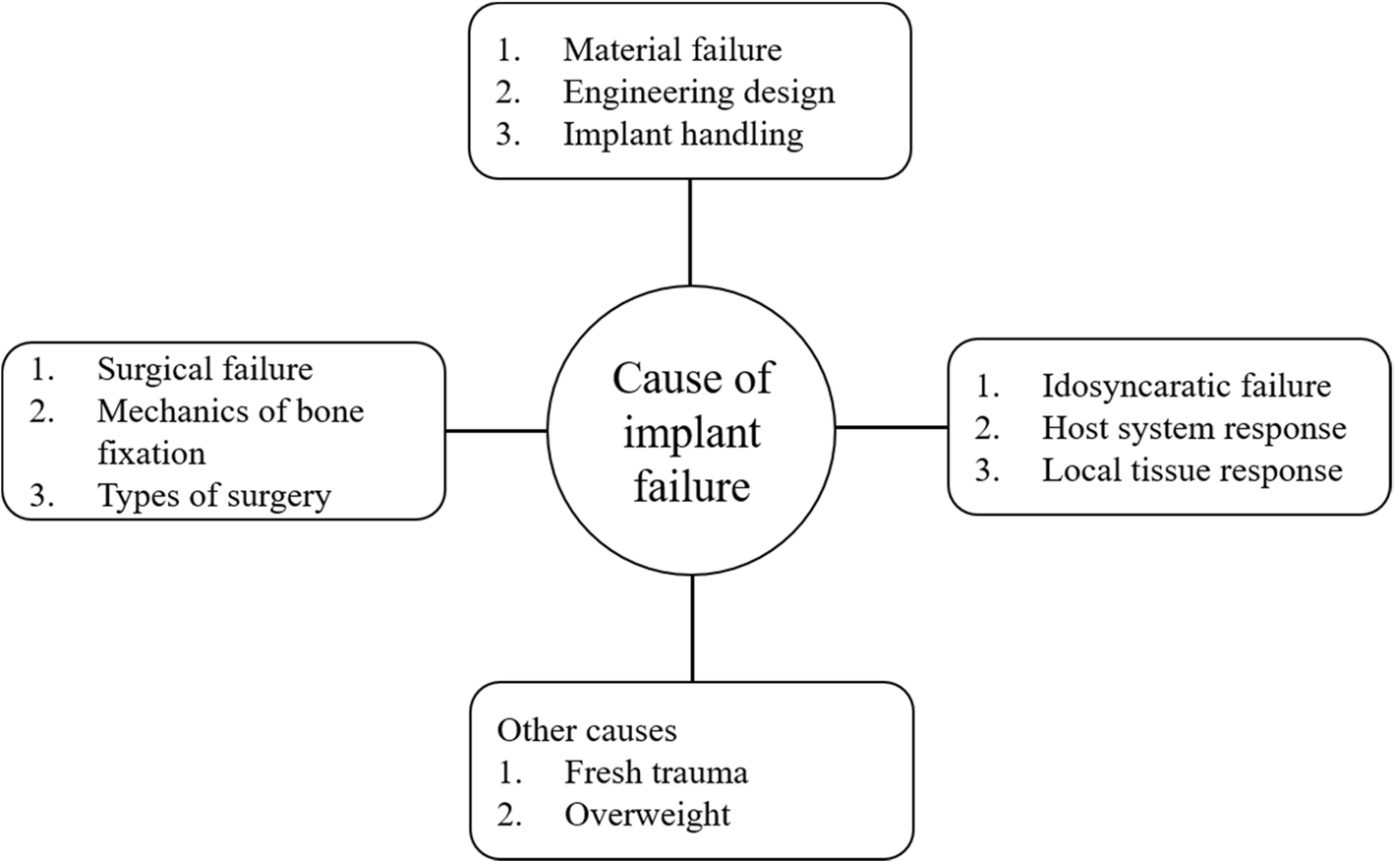
Schematic diagram representing causes of orthopedic implant failure

According to the American Census Bureau, the population over the age of 65 will increase by 53.2% by 2020 [16, 24]. This ratio is increasing every year along with a growing number of bone-related diseases, demanding numerous procedures and innovative techniques. These bone-related problems affect millions of the people every year, with the majority above 65 years old. Figure 1 shows that, along with the host immune system (diseases and obesity), improper handling of implants, surgical techniques and the operating room environment are the major causes of implant failure [25]. Two major implant-related problems are outlined below: septic and aseptic loosening of prosthetic components and implant coating to enhance osseointegration sections.

### Septic and aseptic loosening of prosthetic components

Prosthetic joint replacement (PJR) failures due to loosening is a crucial issue that arises for different reasons. Septic and Aseptic loosening are two distinct conditions with few things in common. One of the common phenomena contributing to both types of loosening is the mechanism that activates the macrophage. Here, septic loosening is caused by virulent bacteria like *S. aureus*, bacteria that come in contact during surgical procedure causes acute postoperative inflammation resulting to periprosthetic bone loss. The symptom of acute septic loosening is common (fever and chills) this makes the diagnosis difficult at initial phase [26]. The cause behind the septic loosening of PJR is mainly due to the rapid development of acute infection of the artificial implant by contagious bacteria [27]. Other reasons for septic loosening are prosthetic bone loss caused by bacterial-induced inflammation and mechanical dislodgement of the prosthetic underlining bone bed [28]. Septic loosening causes an increased ratio of early infection-producing symptoms including pain, functional disruption, redness, fever and purulent drain from the surgical site.

Aseptic loosening is slow process that develops over years. In early days symptoms are mostly absent and diagnosis is done over routine follow-up. The loosening is initially driven by low-grade biomaterial wear debris produced from the bone cement, implant surface [29]. Aseptic loosening of PJR, initially known as ‘cement disease’ [30], is a gradual process that usually takes a long time. In the early phase of implantation, symptoms are almost absent as the problem is only evident during routine radiographic diagnosis, which reveals light wear of the load bearing implants and growth of osteocytes lesions. This condition is primarily driven by early inflammation caused by wear fragments freed from the load bearing surface and the boundary between bone cement or bone, Poly methyl methacrylate (PMMA) debris and polyethene particles from Ultra-high-molecular-weight polyethylene (UHMWPE) implants [31, 32]. Surgical implant failure due to aseptic loosening has been a major problem with increasing ratios caused by biological and implant fragments, affecting bone resorption (inflammatory cell influx) and loss of prosthetic support. Approximately 25% of prosthetic revision surgery is due to aseptic loosening [33] and 28–29% of cemented implant failures are also due to the repetitive mechanical stress associated with locomotion [34]. For non-cemented implants, aseptic loosening occurs due to the degree of osseointegration between bone implants.

### Implant coating to enhance osseointegration

Bone-implant attachment under a normal state clinically reproduces osseointegration, in conjunction with improvements in the structural and functional connection of bone implants. The regenerated bone connection to the implant exhibits an increase in mechanical stability. An occurrence of osseointegration follows a similar mechanism to bone fracture healing with direct contact between bone and implants [35]. When placed in the host body an implant device forms an inert oxide layer hindering bone-implant interaction [36]. This results in the ultimate failure of an implant due to insufficient integration into the surrounding tissue. From the early 1990s until now much research work has been carried out to combat osseointegration and implant loosening along with infection diminution. Usually, to overcome the issue, clinical implants are coated with a bioactive matrix which has given promising result in bone tissue integration. Recently published research work addressing osseointegration with the abundantly practiced coating matrixes to enhance biocompatibility and bioactivity alongside reducing implant infections is discussed in Table 1.

**Table 1:** Some of the most recent and widely practiced coating techniques used to enhance osseointegration with experimental finding

**Coating type**	**Techniques and materials**	**Effective for Osseointegration**	**References**
**Hydroxyapatite (HA) Coating**	In situ observance for 7 days, 20–30 µm Hydroxyapatite (HA) coating on bifunctional Ti-implant	Prevention of bacterial growth in an inoculated medium, enhanced adhesion, cell proliferation, and osteogenic differentiation	Liu et al. [37], 2018
	In vitro and in vivo study for osteogenesis effect of strontium-substituted HA coating, 12 weeks observation on rabbit radial	10% SrHA coating inspires osteogenesis, effective bone regeneration biomaterial	Li et al. [38], 2017
	In vitro experiment on rabbit femora, observed for 12 weeks	Demonstrated enhanced osseointegration, improved antimicrobial properties	Woźniak et al. [39], 2018
	In vivo experiment conducted to identify the bone-implant interface and efficacy of electronically deposited HA coating on the interfacial osseointegration	Significant improvement in early-stage osseointegration and enhanced bone-implant bonding	Lu et al. [40], 2020
	In vivo experiment conducted on rabbit model and *in-vitro* study conducted by coating strontium-substituted HA (SrHA) on Ti-implant	Both the in vivo and in vitro experiments showed this SrHA coating promotes osteoblast growth and osteogenesis along with osteoclastogenesis	Geng et al. [41], 2021
**Extracellular Matrix (ECM) coating**	ECM used as a surface modification of orthopedic implants	Ti-implant is coated with ECM, which improves new bone formation. Enhanced bone-implant interaction	Zhao et al. [42], 2013
	Innovative bone-derived Titanium-coating with ECM bone matrix components (type I collagen), implanted in the distal femur of a white rabbit. Comparing coated and uncoated implants for 45 and 90 days	Increased integration by proposed surface coating. Enhance the stable fixation of implants	Cecconi et al. [43],2014
	Ti-implant is coated with ECM proteins	The coated implants increased their hydrophilicity and conclude that the use of ECM visa atmospheric plasma enhances cell adhesion, proliferation	Tan et al. [44], 2019
	Both the in vitro and in vivo evaluation of biomimetic Ti-implant coated with mineralized ECM obtained via bone marrow mesenchymal stromal cell culture	The result concluded that this biomimetic Ti-implant speeds up the osteogenesis of bone marrow stromal cell via cell proliferation	Wu et al. [45], 2020
**C. Magnesium (Mg) coating**	Mg-containing ceramic coating on Ti-implant to reduce the inflammatory response	Effective as anti-inflammatory agents, Mediates osteogenesis	Li et al. [46], 2018
	In vivo analysis of Mg-based bone implant (screw), implanted in goat femoral condyle fracture fixation, studied effect for 18 months	Demonstrates higher osteogenic factor level, promotes the new bone formation	Kong, Wang [47], 2018
	Analysis of antibacterial effect on Ti-implant coated with Mg, placed in the human osteoblast and *S. epidermidis* culture	A promising material for antibacterial action on the implants reduced corrosion ratio	Zaatreh et al. [48], 2017
	In vitro study of the addition of Mg on Ti-implant by micro-arc oxidation method	The samples analyzed by energy-dispersive X-ray spectroscopy demonstrated Mg is well coated in Ti-implant. This nano-coating enhance cell proliferation, osseointegration and cell adhesion	Li et al. [49], 2020
**Chitosan coating**	In vivo study of carboxymethyl chitosan-zinc for prevention of infection in24 male rabbits up to 2-4 weeks	Prevention of early infection, effective in the prevention of pin tract inflammation	Martin et al. [50], 2018
	In vitro analysis of gallium-modified chitosan coating on Ti-implants to enhance the implant function	This process limits the bacterial colonization, adhesion and sustains osseointegration capability	Bonifacio et al. [51], 2018
	The Ti-implant coated with the chitosan Ag and HA composite nano- coating via electrochemical deposition method	This demonstrated the enhanced abilities of antibiosis, osteointegration between the implants and bone	Wang et al. [52], 2019

### Hydroxyapatite coating for osseointegration

Hydroxyapatite (HA: Ca_10_(PO_4_)_6_(OH)_2_) coating on the load-bearing implant was first proposed during the late 1960s and is used as an alternative for cemented fixation due to its natural osteoconductive and bioactive character [53, 54]. Crystalline hydroxyapatite has a three-dimensional geometry [49] and its principal mineral component corresponds with natural bone. This is used as a coating material, it enhances the osteoconductivity, stimulating bone proliferation and the attachment of osteoblast cells on the surface of the implant [55]. The coating of HA on the implant surface increases wear resistance including osseointegration and mechanical enhancement [56, 57]. HA has a substantial in vivo success rate [58, 59] and extended implant lifetime [60]. Research shows that plasma-sprayed HA coating on Ti6Al4V demonstrates direct adhesion of new bone with HA coating and an implant [61, 62]. A hydroxyapatite-coated implant-bone interface is chemically and biologically bonded directly with mark-to-mark new bone formation between the gaps (from 1–2 mm to 400 μm) [63].

Regardless of the long clinical history of HA coatings on implant surfaces, it has had mixed results concerning osseointegration [34]. Bioactive material (HA) coating on the implant surface enhances osteogenesis process by reducing the inflammation, increasing bioactivity that contributes to enhanced osseointegration in bone tissue. Osseointegration depends on biological properties of biomaterials, HA coating layers in the implant surface also enhance the ability to induce string bonding to host bone contributing in osseointegration [64]. For intensified HA performance, researchers have discovered alternative procedures: HA mixed with active biological and pharmacological agents [60] and HA mixed with ceramic [63]. Ti6Al4V scaffolds coated with Polydopamine assisted HA- implanted in rabbits resulted in amplified cell proliferation, improved attachment, and the bioactivity of MC3T3-E1 cells [65]. The study by Yang et al. [66] shows the hydroxyapatite/ phase-transited lysozyme (HA/PTL) multilayer coating on titanium implants both in vivo and in vitro and concluded there was boosted biocompatibility and osteoinductive phenomena. Phase-transited lysozyme-assisted Polyhydroxyalkanoates (PHA) is a simple, rapid, cheaper surface coating technique [66]. Strontium-substituted hydroxyapatite promotes angiogenic factor CD31 along with osteoblastic genes to enable angio-osteogenesis [67]. There is also additional recent research that includes the HA coating with novel mixtures for better results. Woźniak et al. [39] study demonstrated all the rabbits had HA doped silver nanoparticle coated cylindrical implants resulted in improved optimal Osseo-integrative and antimicrobial properties. A carbonated HA coating matrix has outstanding bioactivity and improved wettability expanding protein adsorption [68]. In addition, manipulation of the immune reaction of macrophages can be done by changing the structure of the HA matrix to nano dimensions which can provide a robust foundation for the upcoming design of a surface coating matrix [69].

### Extracellular matrix/ collagen coating

Current interest for improving bone osseointegration largely involves surface coating of implants with the biologically extracted extracellular matrix (ECM). ECM provides support and anchorage for the cell and tissue regeneration. It segregates tissue and regulates intercellular communication. Collagen fibril has the propensity to boost osteoblasts and mesenchymal stem cells increasing subsequent improvised osseointegration and the bone-implant relationship [70]. Pre-coating of immobilized collagen on the implant surface improves the in vivo host acceptance. An implant coated with type 1 collagens enhances osteoblast and osseointegration and Mesenchymal Stem Cell growth mediated through integrin β1 created pathways [71, 72]. Immobilization of orthopaedic implants, either with adsorptive or covalent plasma coatings with cartilage ECM molecules Glycosaminoglycan chondroitin sulfate, increases the effect of the collagen 1 coating [73, 74]. The test implants coated with covalently immobilized type-1 collagen have enhanced cell adherence, cell proliferation, and cell attachment in terms of cytotoxicity.

ECM are biologically extracted which makes them vulnerable to microbes; and implants coated with ECM can elicit infection during implantation. Another drawback is that ECM suffer from substantial batch-to-batch variability in quality due to the biological extraction procedures. Artificial peptide emulating techniques like Arg-Gly-Asp (RGD) are used to eliminate associated problems [75]. Research shows that an RGD coated titanium implant improved osseointegration in several animal studies [76, 77]. Rammelt et al. [78] inserted six titanium rods coated with lyophilized type-1 collagen and other uncoated rods into the tibias of mature male Wistar rats and absorbed lyophilized type 1 collagen under observation for up to 28 days. After 28 days bone regeneration was 76.3% and 67.8% for collagen-coated and uncoated rods respectively [78]. This research indicates improved primary bone regeneration using titanium rods with a collagen coating.

### Magnesium coating

Magnesium and its alloys have high strength and rigidity for the internal retention of bone fragments and are completely absorbable [79] resulting in its numerous applications. These include surface modification, bone repair, and osseointegration phenomenon. In normal adult human weighing 70 kg have nearly half of the total bodily magnesium deposited in bone material which is essential for metabolism [80]. Earlier research in the magnesium coating (MC) have demonstrated that it accelerates hard callous foundation by adhesion of osteoblast and new bone formation [81]. Deficiency of Mg during the implant leads to negative bone mass density [82]. MC-implant add-ons increases the amount of Mg on bone density [83, 84]. Zhai et al. [85] studied magnesium coating on total joint arthroplasty (TJA) which found that Mg has a significant influence over the proliferation and apoptosis of osteoblast and on osteoclast formation. Mg also unveils antifungal and antimicrobial properties against *S. Aureus* which averts bacterial addition on the implant surface and biofilm formation [84]. Magnesium and its alloys were used in the surface coating of porous titanium implants Ti6Al4V by Li et al. [86] to improve the osseointegration of Ti. The in vitro study shows suitable biocompatibility and biodegradable properties of the magnesium coated titanium implant (MCTI). The non-cytotoxicity behavior boosted MC3T3-E1 cell proliferation. The author summarized that MCTI promotes bone regeneration and better osseointegration in rabbit femoral condylar was observed after 4 to 8 weeks in comparison with uncoated Ti. The release of Mg coated on implant surface passages to the periosteal region via Harversian or Volkmann’s canals was enhanced as the diameter of Mg ions are much smaller than those canals i.e. (< 300 pm) [87]. Therefore, the MCI can comparatively enhance the bone regeneration and reduction of biofilm formation. Thus, MCI resists corrosion and amplifies biocompatibility with an antibacterial effect in vitro with enhanced osteogenesis and osseointegration properties compared with uncoated titanium implant.

### Chitosan coating

Chitosan is synthesized from natural renewal polymer chitin from deacetylation in an alkaline media [88]. It is a highly biocompatible and biodegradable polymer with numerous areas of application, one of which is the surface coating of orthopedic implants. The primary function of chitosan is antimicrobial, because of its poly-cationic nature and antifungal enhancement in osseointegration, even speeding up wound healing [89, 90]. Chitosan is non-toxic, biocompatible and bio-adhesive with unstable biomolecules this makes chitosan a valuable component in formulation of drug. Hence, it’s also used as an antitumor, immunoadjuvant and is anticholesteremic [91]. An antimicrobial function of the chitosan-coated implant is facilitated by electrostatic force among the protonated amino groups (NH_2_) in chitosan and negative residues in the cell surface [92]. Currently, post-surgical prevention search is widely carried out. D’Almeida et al. [93] studied antibacterial action against *Escherichia coli* and *Staphylococcus aureus* strains in an animal-free chitosan -embedded titanium alloy implant. Thus, the immobilized chitosan success rates can be identified via surface characterization techniques and enhanced bacterial effects. Chitosan-coated on titanium screw indicates reduced infection ratio and healing sequence of woven bone formation, fibrous followed by the formation of lamellar bone [94]. A Ti implant coated with chitosan in vitro demonstrated prevention of staphylococcus epidermidis ATCC 35,984 and biofilm formation [95].

### Future directions for enhancing osseointegration

Future research demands more emphasis on fabrication and surface modification procedures. Those procedures should have a higher ability to discretely control biological, chemical and physical phenomena after being implemented in a host**.** Ionization of the implant materials causes a reaction with the biological host system resulting in bone nonunion, implant loosening and weaker osseointegration. These advanced techniques will be applied to the development of implant surface coatings to develop control of biomolecules. This will also intensify the ability of coating techniques to prevent delivery of bioactive biomolecules. Implant coating should be carried at the basic level to modulate acute inflammation, prevent chronic infection, stimulate osseointegration and at the same time induce the reparative stage. As a crucial issue in implant surgery, osseointegration demands more research focusing on surrounding bone growth, with the optimal design of the biomaterial porous surface to encourage bone ingrowth and implant stabilization. Other potential studies can involve the investigation of novel biomaterials and polymer coating techniques which can enhance bone regeneration and even the interaction with host cells in predicted mode rather than just replacement.

### Implant coating to reduce bacterial infection

Systematic antibiotic prophylaxis has always been the most common strategy to avert early implant-related infection. This process is not effective in delayed or late infections with a timeline extending to years making it difficult to identify the infection and eradication of biofilm. This increase in the infection ratio due to antibiotic-resistance reinforces the need for active, preventive solutions. The resolution for this condition can be obtained by a change in the bulk properties of implant material that hinders bacterial adherence. This could be implementation of surface coating techniques preventing adhesion, colonization and biofilm formation. The socio-economic time frame of surface coating techniques provides a favorable immune cell response and biocompatibility [96]. Passive antifouling surface coating, super-hydrophobic structuring and smart polymer coatings are frequently used surface coating techniques to avert bacterial cell-surface collaboration. Direct coating of antibiotics to the implant surface and antibiotic loaded matrixes have been clinically used [97]. A significant amount of research has been carried out in search of effective treatments for implant infection to resist biofilm [12]. In the present context, many coating techniques with quorum-sensing quenchers, antibiotic-antimicrobial coatings and host immune modulator coating are in use. Some of the techniques for antibacterial coating on implants are included in this review below in Table 2.

#### A. The direct Antibacterial/antimicrobial coating

The customary method to reduce implant infection and biofilm formation is to use an antibacterial coating on the implant surface. Systemic antibiotic prophylaxis is consistently applied especially for the prevention of postsurgical infection [107]. However, systematic drug administration is relatively low for target delivery and impending toxicity as a skeletal system has poor vascularity. To inhibit bacteria, antibiotics are locally/directly used in implant surgical site in higher concentration [108, 97]. During surgical closure, antibiotics in powder form, such as vancomycin, are directly sprinkled on the incision to reduce the Surgical site infection (SSI) ratio during tibia plateau, spinal deformity and fracture fixation [108]. This shows reduced infection rates with minimal local and systemic risk in the adult population [109]. The antibiotic prophylaxis in bone cement can help reduce deep infection, revision surgery and aseptic loosening of implants [110].

In conjunction with the above, covalent attachment of antimicrobial peptides provides a defense against non-specific interactions, and diminishes the impact of surface effects and confinement [111]. The ability of covalently bound vancomycin coatings on Ti-implant surfaces to constrain S. aureus and S. epidermidis in vitro and in vivo was demonstrated by Jose, Antoci et al. [112] more than a decade ago. Covalent merged vancomycin with the titanium-alloy implant surface resulted in significant inhibition of *S. epidermidis* biofilm formation [113]. This covalently chained vancomycin showed substantial control of bacterial colonization and amended osseointegration even after 3 months observed in an animal model [114].

A recent publication commented on vancomycin coatings for the reduction of implant-associated infection with novel electrostatic dry powder outlined, release and effects observed both *in-vivo* and *in-vitro* for 7 days. It specified biocompatibility for the osteoblast cell line MG-63 together with higher antibacterial ability against methicillin resistance *S. aureus* (MRSA) [115]. Gentamicin is another commonly used antibiotic for the reduction of implant infection [116, 117]. Other antibiotics with broad spectra, like amoxicillin, cephalothin, tobramycin, and carbenicillin are used as implant coating drugs [117, 118]. For controlled release, surface coating of implants with drugs like tobramycin, cefamandole, rifampicin or gentamicin is in wide use [117]. Direct coating of antibiotics on implants leads to burst and instant release 80–90% within the first few hours [119]. To increase the sustained release of drugs for a longer period, they can be incorporated into the matrix or hydrogel with controlled pore size. Recently, covalent coating of the drug onto the surface of an implant has been trending for sustained release. The titanium implant surface is covalently modified by aminopropylation which is extended by tethering solid phase coupling of ethylene glycol linkers, this is further followed by phase coupling of vancomycin. Vancomycin now is successfully covalently bound in a titanium implant surface preventing bacterial adherence and organized release [120]. This can be advantageous for reinforcement of antibacterial capacity on the implant surface while concurrently eradicating the side effects of burst release of drugs in body fluids.

#### B. Antibiotic-loaded Hydrogel coating on implants surface

**Table 2:** Recent commonly used coating techniques to combat inflammation, bacterial colonization, and biofilm formation and their experimental findings

Coating type	Techniques and Materials	Effective for Antibacterial	References
**Covalent coating of Antibacterial/Antimicrobial coating**	*In-vivo* study of covalent coupling of antimicrobial on Ti-implant surface implanted in mice	Reduction of implant-associated inflammation, enhance cell proliferation and osseointegration	Gerits et al. [98], 2016
	*In-vivo *analysis of covalent immobilization of antimicrobial on Ti-implant for the prevention of biofilm formation	Significate reduction of bacterial colonization, enhanced osseointegration both in vitro and in vivo	Kucharíková et al. [99], 2016
	Beta-tricalcium (β-TCP) phosphate samples loaded with rifampicin form II and produced in powder form	The antibacterial efficacy against *S.aureus* is significantly enhanced along with biological performance and compatibility	Topsakal et al. [100], 2020
**Antibiotic-loaded Hydrogel coating**	Clinical analysis of the antibiotic-loaded fast resorbable hydrogel on in closed fracture fixation procedure for 253 patients	Reduce post-surgical site infection, speeds up wound healing	Malizos et al. [101], 2017
	*In-vivo* study to identify the ability of Defensive Antibacterial Coating (DAC) of implants for the prevention of acute bacterial colonization, 30 rabbits observed for 7 days	Vancomycin loaded DAC: prevent infection in the implant site without any side effects	Giavaresi et al. [102], 2014
	Both in vivo (in mice) and in vitro study of moxifloxacin (A50) sol–gel with variable antibiotic concentration to prevent bacterial infection in prosthetic joint	The greater concentration of moxifloxacin (A50) demonstrated excellent bactericidal and anti-biofilm response with greater inhibitory effect. Significantly effective against *S. aureus, E. coli* and *S. epidermidis*	Aguilera-Correa et al. [103], 2020
	Clinical analysis of cementless prosthetic implants coated with antibiotic loaded hydrogel (ALH). The human sample size is 17	The study shows ALH effectively reduces the infection in prosthetic joint. No significant difference observed in function and prosthetic osseointegration	De Meo. et al. [104], 2020
**Silver antimicrobial coating**	An *in-vitro* study of nano-Ag-loaded coating on Ti-implant to analyse the biological performance of the coating	Inhibits bacterial colonization, enhances the proliferation and cell growth around the implant site	Zhang et al. [105], 2018
	The study evaluates the effectivity of antimicrobial multilayer silver coating techniques that includes the in vivo experiment in which rabbits have methicillin-sensitive *S.epidermidis* (MSSE) coated in K-wire inserted for 7 days and are scarified for clinical analysis	With the significant enhancement in bacterial inhibition, silver multilayer-coated (SML) implants were free of pathogens and no silver was detected in blood proving the SML coating more effective in combating bacterial infection in implants	Fabritius et al. [106], 2020

Hydrogels have both hydrophobic and hydrophilic character and are biocompatible which can be refilled without revision surgery over time. These types of hydrogels give sustained drug delivery over time and control surgical site infection related to implants [121–123]. They are widely used for implant coatings as a measure to reduce infections related to implants and for prevention of implant failure. The smart hydrogel, responsive to pH and temperature is further complemented by its highly biocompatible and biodegradable characteristics. Zhai et al. [85] proposed a fast resorbable antibiotic-loaded hydrogel coating on an implant surface to prevent post-surgical infection and for osteosynthesis [85]. This is also known as a defensive antibacterial coating (DAC), it consists of covalently linked hyaluronan and poly-D, L-lactide which is designed to undergo complete hydrolytic degradation in vivo*.*

In the study, 256 patients who underwent osteosynthesis for closed fracture fixation were allotted DAC for approximately 18 months. It was observed that it can significantly reduce post-surgical infection on the implant site. Along with this, there is much other research concerning DAC coatings on implant surfaces to augment osseointegration and for diminution of SSI. Drago, Boot [124] coated antibacterial (gentamicin, amikacin, tobramycin, vancomycin NAC) loaded hydrogel on an implant surface [2] and significant effects were observed. Surface coating of implants with fast-resorbable antibiotic-loaded hydrogel has a noteworthy fail ratio of early SSI [100] observed from a clinical trial. DAC hydrogel coating is biocompatible and does not interfere with implant osseointegration [125]. DAC loaded with 2% of w/v vancomycin was coated on intra-medullary nails which were ultimately used for femur fixation of an adult New Zealand rabbit showing reduced bacterial colonization in an animal model with highly loaded bacterial contamination of an implant [101].

**Fig. 2:**
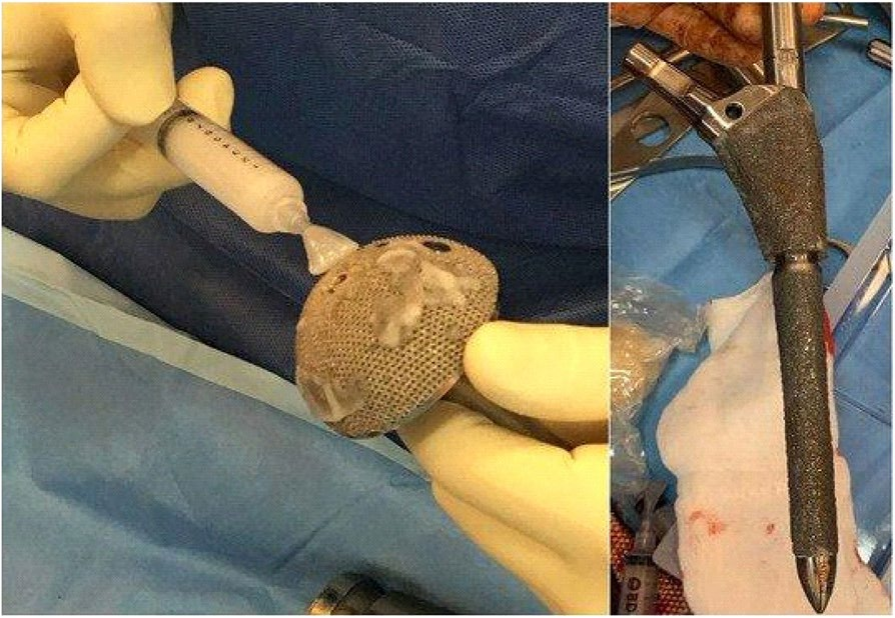
Experiment on “Antibacterial loaded hydrogel coating on final implant” De Meo. et al. [98]

Figure 2 illustrates the experiment on “Antibacterial loaded hydrogel coating on final implant” De Meo, et al. [104] reveals that an implant coated with an antibiotic has a significant effect on bacterial inhibition. The implants were coated immediately before the insertion and divided into two groups. 1). Antibiotic loaded hydrogel (ALH) insertion with 5 ml of hydrogel mixed with 200 mg of gentamicin total of 14 patients; and 2). Dual antibiotic loaded hydrogel with 250 mg of vancomycin mixed with 5 ml of hydrogel and 200 mg of gentamicin in four patients [104].

Commercially applied Poly (D, L-lactic acid) (PDLLA) loaded with gentamicin used for the surface coating of implants revealed inhibition of bacterial colonization. Rapid release of the drug initially was followed by a sustained release for about a week where PDLLA degraded in nearly six months [126]. The ability of the hydrogel to adhere to an implant surface provides corrosion resistance. Related publications regarding surface coating of an implant with antibiotic-loaded biodegradable hydrogel are abundant. However, this is not commercially practiced [127, 128].

A thin layer implant coating with poly (N-isopropyl acrylamide) (PNIPAM) hydrogel diminishes chronic inflammation on the implant sites with an increased level of macrophage 80% [129]. PNIPAM hydrogel consists of PNIPAM-co-AA microgel particles which are crosslinked with polyethylene glycol (PEG) diacrylate tethered onto a polyethylene terephthalate substrate. Copolymer based hydrogel loaded with a ciprofloxacin coating on a titanium implant was used for testing in vitro methicillin resistance *S. aureus* (MRSA) where MG63 osteoblast cells assess the biocompatibility of ciprofloxacin loaded hydrogel coatings [130].

#### Silver antimicrobial coating

Silver (Ag) has been used as an antimicrobial element for centuries. Numerous studies have investigated silver as a promising antimicrobial coating material [131, 132]. The silver coating on suturing wire has been used from an early time. It is widely used in urinary catheters and central venous catheter coatings with a significant reduction in inflammation [133, 134]. Different techniques are used for silver coating, varying in chemistry, loading amount, release pattern and mechanism of the matrix. Ag inhibits gram-positive and gram-negative bacteria and offers long-term effects.

The mechanism of the silver ions also disrupts the cell membranes of bacteria, the metabolism, and formation of DNA [135]. Silver ions bind with the thiol group in a bacterial membrane and metabolize the enzymes [136]. This disruption of the bacterial respiratory enzymes damages the cell membrane disabling the bacteria protecting protein assembly. The surface of the implant is coated with silver ions. These silver ions physiologically bind with the host ions (Chlorine, Sulphur), reducing toxicity to the host system with increased antimicrobial efficiency. When using silver ions alone in the coating will result in bacterial colonization control. However, a silver coating on the surface of the implant with the drug (daptomycin and vancomycin) separately demonstrated major preventive significance [131]. The same study shows that the dual drug combination has 100% preventive results. The known negative of silver coating is burst release giving a systematic effect and local toxicity [137] information regarding long-term tissue toxicity. More research has published on silver coatings for titanium screws was concluded that this can prevent deep bone infection when anodically polarized [138].

### Future trends for the control of infection

There is a significant demand for a detailed study of the physical constraints that employs an advance and sustained approach. This will facilitate objective evaluations between distinct surfaces in both natural and reformed procedures. However, this gap in understanding can only be narrowed through the improvement of strategies for highly controlled modification of implant materials. Ionization of biomaterial is found to trigger infection in many cases, which corroborates the demand for the improvement of the materials used in manufacturing implants.

Aimed at *in-vivo* study of anti-adhesive surfaces that can prevent bacteria and intensify host cell attachment, this could lead to enhancement of tissue integration. For these reasons, formulation of test conditions that mimic the in vivo environment could be considered more relevant for clinical applications. Regarding the biofilm, early infections are not easily diagnosed. Future research can pave the way for detection techniques that could identify polysaccharides or other unique components in the biofilm. It would not only benefit laboratories to be able to identify species-specifications involved in biofilm. There would be a profound impact on patients by reducing both diagnosis and treatment duration. This could reduce the economic burden of healthcare.

### Commonly practiced surface modification techniques

Orthopedic implant-related surgery is considered a success when the implant has a stable fixation and minimum bacterial infection. To fulfil the increased demand for implant surgery and accelerate the osseointegration process, various implant materials have been selected that offer excellent functional properties, like stainless steel, titanium and its alloys, cobalt-chromium and its alloys, zirconia and polymers. However, along with the coating materials (natural and synthetic) discussed above, surface modification techniques are used to further enhance the functional and mechanical abilities of these materials. These techniques reduce the possibility of inflammation, and enhance corrosion resistance, biocompatibility and modulus of elasticity of substrate [139]. Surface oxidation, wear resistance, and implant degradation are initiated on the surface. Hence external stimuli are used on the substrate for modification. Some of the commonly used surface modification techniques are Chemical Treatment (CT), Biological Techniques (BT), Plasma Spray Technique, Sol–gel Technique and Texturing.

### Chemical Treatment (CT)

Chemical surface treatment enhances the biocompatibility between the implant and bone by generating similar chemical bonding to that of bone [140]. It is categorized into two groups: specific and non-specific. Examples of a non-specific chemical reaction are oxidation of a polyethene surface by chromic acid and radiofrequency glow discharge (RFGD) plasma treatment. The specific chemical surface reaction occurs when a solo functional group is converted into another with a high yield and side reaction, for example, alkylations and alteration of siloxane, Filler et al. [141]. Inside this chemical technique, numerous methods are followed for surface modification including alkali treatment, hydrogen peroxide treatment and acid treatment.

### Biological Techniques (BT)

Biological coating techniques are practiced both in vivo and in vitro experiments. Techniques, like cell seeding and natural coatings promote cell proliferation, osteoblast adherence, and cell differentiation. On the surface of the porous implant, different cells and proteins have been seeded [142, 143]. However, the efficacy of this method depends on the differential potential of cells, density, position and implant design [144].

### Plasma spray technique

Plasma spraying (PS) is the only coating technique practiced clinically. Here the sample coating materials are loaded on a plasma jet. These samples are melted using the thermal heating technique and coated on implants under the plasma torch creating a vacuum. PS is a cost-effective and safe procedure [145]. Hydroxyapatite (HA) is a commonly used coating material in PS. It has an excellent deposition rate and compact layer formation compared to other techniques Singh et al. [146]. The HA coatings on the implants using this technique resulted in enhanced corrosion resistance and bioactivity of metallic substrate Fazel et al. [147].

### Sol–gel technique

Sol–gel technology is a simple wet-chemical method that creates an oxide layer changing the pH of the implant surface or, with the sol-gelation method, by thermal treatment. This process changes the solution to aerogel or ceramic with altered guidance as necessary. The major advantage of the sol–gel surface modification technique is that it utilizes a low-thermal heating technique allowing first-rate control over the chemical coating. It is also used in drug-loaded hydrogel coatings with a highly controlled release rate [148].

**Fig. 3:**
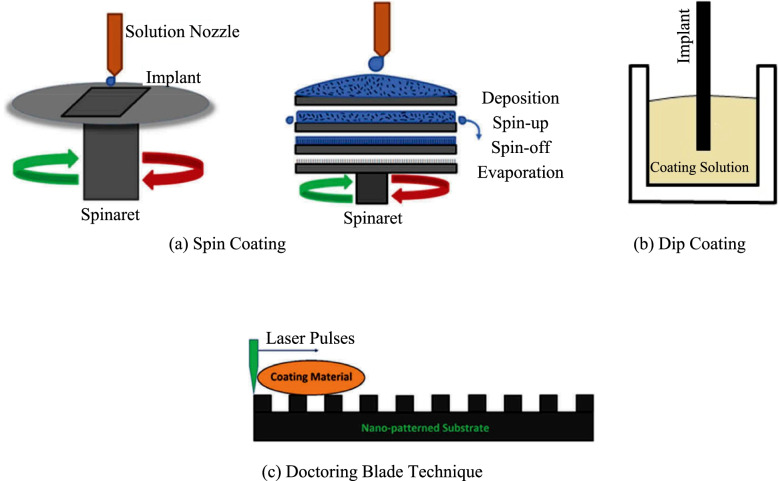
Types of sol–gel coating techniques on implant surface Priyadarshini, Rama et al. [149]

The Fig. 3 above demonstrates the simple steps followed in the surface coating using the sol-gel coating method, liquid immersion and electrophoretic process. The sol-gel process is broadly used in thin coating (< 10 µm) ceramic coating [150].

### Texturing

The process of texturing modifies the surface topography of the implant surface by creating microspores and microchannels. This method is intended to facilitate elastohydrodynamic lubrication to reduce the frictional forces between the mating parts. Texturing improves the surface area, the strength of the implant and decreases surface scratch risks. Etsion et al. [151] demonstrated how microscale and nanoscale textures contributed to cell interactions on the implant surface and regulates cell proliferation, signaling and adhesion. The combined effect of hydrophobic or hydrophilic configuration or capillary force might be responsible for the self-organization of protein molecules and cell attachment Kurella et al. [139]. Various surface texturing methods using a surface modification are listed in Table 3.

**Table 3:** Various surface texturing methods using a surface modification

Texturing Process	Features
**Sandblasting** Martin et al. [152]	A random surface texturing process, difficult to control the depth and regularity of the substrates
**Electron beam texturing** Rajnicek et al. [153]	Precise control: requires a vacuum
**Photolithography** Clark et al. [154]	Demonstrates well-controlled features, the mutual problems with organic solvent, spin coaters and photoresists process
**Electric arc texturing** Curtis et al. [155]	Used for conductive materials: lower control over the process
**Laser texturing/ micromachining** Duncan et al. [156]	This process delivers precise control of even complex features: fast, clean and no contact

The physical surface modification technique is like a grit blasting technique that uses rough particles. TiO2, HA or alumina is used in the implant surface by applying force and pressure of compressed air [157]. This method cleans the residuary particles while accelerating osteogenesis [158]. In addition, a new and promising technology known as additive manufacturing (AM), also commonly referred to as 3D printing/rapid prototyping, is being used more widely. Substrates are modified while manufacture in a layer-by-layer fabrication method selectively melting by laser and electron beams [157]. The substrates which undergo AM modelling are clinically relevant with increased mechanical strength contributing enhanced collagen deposition and adhesion of mesenchymal cells [159].

## Discussion and conclusion

Implant surgery to combat functional and physiological characteristics has been trending for a long time for orthopedic applications. However, it suffers from diverse complications. This has advanced the treatment procedure. While this includes the invention of new materials and alloys with higher biocompatibility, mechanical and functional strength, surface modifications and implant coating techniques have also been developed. Implant coating has demonstrated outstanding results in vitro and in-vivo. Nevertheless, the complete eradication of implant-associated complications is still not complete.

There is an increased demand for orthopedic implant surgery. However, there are several applications which increase the potential of implant failure due to infection, bone-nonunion, aseptic loosening and osseointegration. This not only gives a negative impact to the emotional and physiological condition of patients but also increases the economic burden of many researchers who have been interested in number of coating techniques. These facilitate implant insertion, reduce infection, enhance biocompatibility, extend the lifetime of the implant and prevent associated complications. Aseptic loosening due to the disintegration of an implant and eventual wear of implants with continuous movement has been major problem. When it comes to augmenting the implant function and adherence, enhancement of osseointegration is an important issue. It encourages the development of optimized and advanced coating techniques to boost implant-tissue integration. Along with the naturally extracted coating matrix, research has also focused on primary proteins, growth factors, and biomolecules to use as the coating adhesive. While focusing on the application of the coating matrix, it is mandatory to have a brief systematic analysis to demonstrate the benefits, biocompatibility, toxicity (both local and systemic), biodegradability and released sustainability. Regardless of the natural or synthetic coating matrix, materials that embrace a higher degree of biocompatibility and biodegradability offer substantial value. Another specific benchmark for the design of a coating matrix involves the ability to activate osteoconductive actions and reduce infection. However, this should not elicit immune or foreign body responses and must encompass antibacterial properties.

Reductive and preventive coating techniques applied either directly, or antimicrobial loaded hydrogel have shown significant results. A combination of the natural or systematic matrix and incorporation of antimicrobial agents has produced promising results in device-related infection control. However, antibacterial resistance has become an important concern, as there may be poor control over drug release patterns (burst or uncontrolled release). For the present therefore, coating techniques that can be effective in the anticipation or disruption of bacterial colonization are of prime interest. This can be further enhanced to improve anti-quorum-sensing agents with the ability to diminish interference with the biofilm.

Existing coating techniques have been shown to be effective in vitro. However, when it comes to clinical practice, few of them are commercially viable. This shows that the ideal coating material must satisfy all the criteria, including mechanical integrity, sustained-release kinematics and host toxicity. Surgeons must be pre-informed about the possible pros and cons of the coating matrix and techniques. Any innovative coating matrix developed, must be able to overcome current issues such as bacterial resistance growth, the porosity of the matrix for sustained release, resorption, and enhanced osseointegration performance. The synergistic combination of the present coating matrix HA, chitosan and collagen with the other biomolecules will help enhance bioactivity and reduce early problems associated with the coating. The combination of antibiotic and antimicrobial use together with the matrix will enhances the sustained release pattern and prevent antibiotic resistance. Incorporation of silver and magnesium into the coating matrix with natural hydrogel could reduce any drawbacks. The use of the dual drugs with alternative and sustained release could lead to the next level of coating techniques. However, the coating matrix must be easily reproducible and should not have long-term storage problems.

## Conclusion

This article discussed the numerous implant coating techniques used both in vivo and in vitro to prevent bacterial infection. Includes both natural and synthetic hydrogels with or without loading antibiotics contributing significant enhancement in the implant life and infection control. Following the brief introduction of implant infection and its type, types of bacteria that contributes most to the implant infection, biofilm, types of natural hydrogels and antibiotics, silver antimicrobial coating. Alongside presenting brief future direction in implant coating techniques and possible ideal hydrogel development techniques.

## Data Availability

Not applicable.

## Abbreviations

AJRR: American Joint Replacement Registry
PJR: Prosthetic joint replacement
PMMA: Poly methyl methacrylate
UHMWPE: Ultra-high-molecular-weight polyethylene
HA: Hydroxyapatite
ECM: Extracellular Matrix
Mg: Magnesium
PHA: Polyhydroxyalkanoates
RGD: Arg-Gly-Asp
MC: Magnesium coating
TJA: Total joint arthroplasty
MCTI: Magnesium coated titanium implant
DAC: Defensive Antibacterial Coating
MRSA: Methicillin resistance S. aureus
ALH: Antibiotic loaded hydrogel
PDLLA: Poly (D, L-lactic acid) (PDLLA)
CT: Chemical Treatment
RFGD: Radiofrequency glow discharge
PS: Plasma spraying
AM: Additive manufacturing
